# Protection of transplants against antibody-mediated injuries: from xenotransplantation to allogeneic transplantation, mechanisms and therapeutic insights

**DOI:** 10.3389/fimmu.2022.932242

**Published:** 2022-08-05

**Authors:** Delphine Kervella, Stéphanie Le Bas-Bernardet, Sarah Bruneau, Gilles Blancho

**Affiliations:** ^1^ CHU Nantes, Nantes Université, Néphrologie et Immunologie Clinique, Institut Transplantation Urologie Néphrologie (ITUN), Nantes, France; ^2^ Nantes Université, CHU Nantes, INSERM, Center for Research in Transplantation and Translational Immunology, UMR 1064, ITUN, Nantes, France

**Keywords:** vascularized organ transplantation, antibody mediated rejection (ABMR), accommodation, endothelium, anti-ABO antibodies, anti-HLA antibodies

## Abstract

Long-term allograft survival in allotransplantation, especially in kidney and heart transplantation, is mainly limited by the occurrence of antibody-mediated rejection due to anti-Human Leukocyte Antigen antibodies. These types of rejection are difficult to handle and chronic endothelial damages are often irreversible. In the settings of ABO-incompatible transplantation and xenotransplantation, the presence of antibodies targeting graft antigens is not always associated with rejection. This resistance to antibodies toxicity seems to associate changes in endothelial cells phenotype and modification of the immune response. We describe here these mechanisms with a special focus on endothelial cells resistance to antibodies. Endothelial protection against anti-HLA antibodies has been described *in vitro* and in animal models, but do not seem to be a common feature in immunized allograft recipients. Complement regulation and anti-apoptotic molecules expression appear to be common features in all these settings. Lastly, pharmacological interventions that may promote endothelial cell protection against donor specific antibodies will be described.

## Introduction

Kidney transplantation is the best treatment for patients suffering from end-stage renal disease because it offers to those patients better survival, quality of life and lower economical cost than dialysis (1, 2). However, the transplant community worldwide is facing the issue of organ shortage (3). Early kidney transplant survival has strongly improved thanks to the refinement of immunosuppressive regimens, whereas long-term allograft survival progressed slowly those last decades (4). One of the major hurdles for late allograft survival is the development of an anti-Human Leucocyte Antigen immunization (anti-HLA Antibodies (Ab)), and more specifically against donor HLA (so called donor specific antibodies, DSA) that can cause antibody-mediated rejection (ABMR), strongly impairing allograft survival and responsible for the majority of late allograft losses (5–7). DSA can emerge after transplantation (*de novo* DSA) as a result of insufficient immunosuppression (IS) or non adherence to immunosuppressive therapy, and are strongly associated with a worst allograft survival due to chronic allograft rejection (8, 9). Nearly one third of first transplant recipients without DSA before transplantation (“low-risk” patients) develop *de novo* DSA, highly favored by non-adherence (10). Recipients of a transplant with preformed DSA (present before transplantation) have an impaired allograft survival compared to those without anti-HLA Ab or with non DSA anti-HLA Ab (11, 12). Desensitization strategies have been developed to eliminate those preformed Ab with mixed results but the most common strategy is to avoid a transplant bearing HLA Ag specific of them, thus increasing the waiting time of the recipients (13). Revisions of allocation algorithms try to increase priority for highly sensitized candidates (14).

In vascularized organ transplantation, the transplant endothelium constitutes the first interface between the recipient’s blood and the donor tissue. DSA bind donor HLA antigens expressed at the surface of endothelial cells (ECs) and can induce endothelial damage leading to ABMR by two different innate immune responses: complement cascade activation (complement-dependent-cytotoxicity (CDC)) and antibody-dependent-cell-mediated-cytotoxicity (ADCC) *via* the recruitment of macrophages and NK cells (15). A third mechanism of endothelial damage involves crosslinking of HLA molecules at the surface of ECs by high doses of DSA, leading to the activation of intracellular pathways inducing the proliferation and differentiation of ECs, two phenomena implicated in transplant vasculopathy (16, 17). It is worth noting that 40-60% of patients with ABMR do not have circulating HLA-DSAs (18–20). This could be linked to non HLA auto- and allo-antibodies, missing-self-related NK activation and other mechanisms that are currently being investigated (21).

Concordant data from various teams have underlined a possible protective effect of DSA anti-HLA antibodies *in vitro* on ECs at low titers (17). This absence of deleterious effect of antibodies on their targets at the cellular or organ level is called “accommodation”. The accommodation concept was built up in the xenotransplantation context and was first defined by Platt et al. as a state in which the vascularized organ would survive in the presence of xenogeneic antibodies (XAb) and complement (22). Moreover, in ABO-incompatible (ABOi) transplantation, the rebound of anti-A/B antibodies after transplantation is often not accompanied by features of ABMR and considered as an *in vivo* situation of accommodation. The accommodation concept could thus be defined as the resistance of the endothelium to the aggression of anti-donor antibodies and complement resulting in little or no injury and a stable function of the transplant.

Given the burden of chronic ABMR and its responsibility in late allograft loss and allograft survival, the concept of accommodation should be further explored in order to develop alternative therapeutic strategies (23).

In this review, we outline the features and mechanisms of accommodation in the ABOi and xenotransplantation settings before describing the phenomenon in the context of allo-immunization and delineating perspectives. We will not develop in this review non-HLA antibodies (auto-antibodies and anti-endothelial cells antibodies) in allotransplantation.

## Observations of resistance to Ab toxicity

### ABO incompatible transplantation

ABO-incompatibility was an absolute contraindication to transplantation until the 1980s. The first ABOi kidney transplantation was performed in 1981 accidentally, with a blood group mistake discovered after surgery, by Pr G. Alexandre in Brussels. Despite one rejection crisis, that could be reversed, the patient went well with a prolonged graft survival. Encouraged by this favorable evolution, further transplants were performed with various results. Most reported cases without desensitization resulted in rapid transplant damage and loss owing to ABMR (24). Utilization of ABOi kidney transplantation with recipient desensitization (i.e. clearance of anti-blood group antibodies) began in the 1980s in Japan, since deceased donor transplantation was not performed due to religious reasons, with such an improvement that it is routinely performed now. ABOi transplantation may increase the number of living donors by one-third (25).

ABO antigens are oligosaccharides expressed on red blood cells, but also on ECs and kidney parenchymal cells (26). In addition to the binding of these oligosaccharides to membrane proteins, variable amounts of A or B epitopes are covalently linked to von Willebrand factor (vWf), a large circulating protein that is released by both platelets and ECs (27). The expression level of ABO antigens varies between individuals. The particular A2 subtype is characterized by a low expression of A antigen and thus a lower immunological risk of ABOi transplantation than other blood-type individuals (28). Preformed isohaemagglutinins are natural antibodies against blood group antigens, induced without immunization with the antigen (29). Their isotypes are mainly IgM but also IgG and IgA, their distribution varying between individuals. The importance of a class over another in acute antibody-mediated rejection of ABOi transplants is unclear. To avoid hyperacute rejection, ABOi transplantation programs include a phase of recipient desensitization to lower the titer of anti-A/B antibodies before transplantation. Desensitization protocols combine the use of apheresis for transient anti-A/B removal, B-cell targeted therapy, long-term immunosuppressive regimen and monitoring of anti-A/B antibodies before and after transplantation (30). Long-term transplant and patient survival after ABOi transplantation are now comparable to those of ABO-compatible kidney transplantation, although some studies highlight an increased risk of early transplant loss or decreased early patient survival due to infections (31–33).

After transplantation, anti-A/B antibody titers that have been decreased by desensitization tend to rebound. Assays and thresholds for these antibody titers vary between centers, as well as the use of apheresis after transplantation when antibodies rebound. In some studies anti-A/B antibody titers after transplantation are associated with ABMR, whereas other studies did not show any relation between these titers and ABMR or graft survival (34–37). Tobian et al. showed a correlation between a rebound of anti-A/B antibody titers (≥ 1:64) and occurrence of ABMR in 46 ABOi kidney transplant recipients after a desensitization protocol including plasmapheresis with or without splenectomy or rituximab (35). On the other hand, Ishida et al. reported the outcomes of 191 ABOi kidney transplant recipients that underwent desensitization before transplantation but did not receive a specific treatment for posttransplant elevated anti-blood type antibodies. They did not find a difference in the incidence of ABMR between patients with low rebound of anti-blood type antibodies (≤ 1:32) and the group with high rebound (≥ 1:64) (37). Interestingly, C4d staining on ABOi transplant protocol biopsies does not seem to be systematically associated with ABMR (38, 39). C4d is a split product from the classical pathway of complement activation. In ABO-compatible kidney transplantation, positive C4d staining in peritubular capillaries is associated with ABMR, reflecting the activation of the classical pathway of complement cascade by anti-donor antibodies. Haas et al. described a positive C4d staining in peritubular capillaries without any correlation with histologic changes suggestive of ABMR in 80% of protocol kidney biopsies performed during the first year posttransplant in ABOi transplant recipients (38). Furthermore, diffuse peritubular capillary C4d deposition on protocol biopsy during the first 6 months was associated with fewer chronic lesions at one year compared to recipients with initial negative C4d staining (40). Bröcker et al. assessed acute and chronic endothelial damage using electron microscopy in biopsies from ABOi kidney transplants (41). They found no evidence of complement-mediated endothelial injury in this context of diffuse C4d staining. Transcriptomic and immunohistochemistry analysis of kidney transplant biopsies from ABOi recipients revealed an upregulation of CD59 (a negative regulator of the membrane attack complex, the terminal product of the complement cascade) at three months posttransplant in C4d positive biopsies (42, 43). Thus, the rebound of anti-A/B antibodies after ABOi transplantation is associated with microvascular C4d deposition, reflecting an activation of complement by anti-A/B antibodies without cellular damage, suggesting a phenomenon of accommodation.

Mechanisms of accommodation in the ABOi setting seem to involve molecular changes in the endothelial cell and changes in the immune response. *In vitro*, high titers of anti-A/B antibodies induce the expression of complement regulatory proteins CD55 and CD59 by ECs, thus inhibiting the complement cascade and complement-dependent endothelial damage (44). This is abolished by co-incubation of ECs with complement and thrombin, suggesting that this direct effect of anti-A/B Ab may not be sufficient *in vivo* for accommodation induction (45). In a mouse model of ABOi heart transplantation, adding complement blockade with an anti-C5 Ab to a conventional triple immunosuppression significantly increased allograft survival despite persistence of high titers of anti-A Abs, with evidence of IgG and C4d deposition but fewer features of endothelial cell injury and vascular inflammation. Moreover, heart transplants from the combination therapy group displayed lower infiltration of T cells, B cells and macrophages. Interestingly, they observed changes in cellular responses with significant reduction of activated T cells and follicular helper T cells in the spleen in the combination group and an increase of CD24+IL-10+ B cells (46). Park et al. studied 16 ABOi living donor kidney transplant recipients (34). They defined accommodation at one year posttransplant by four mandatory criteria: 1) detection of anti-A/B antibodies in the recipient’s serum, 2) normal histology, 3) persistence of A/B antigen in the kidney and 4) glomerular filtration rate superior to 45 mL/min/1,73m². Comparing intra-graft gene expression in four of these ABOi accommodated transplants with five ABO compatible transplants at one year, they found significant differences in the expression of several genes, such as a downregulation of TNF-α and TGF-β1 regulating protein SMAD and upregulation of immunoregulator Muc1 in ABOi recipients. Immunohistochemistry analyses showed a strong expression of Muc1 protein along the glomerular capillary wall in accommodated ABOi grafts, whereas HO-1, Bcl-2, Bcl-xL and Bax were not detectable. These results evoke the dysregulation of the signal transduction machinery and immune surveillance, consistent with the promotion of cell survival. Jeon et al. performed RNA sequencing on peripheral blood mononuclear cells (PBMCs) of ABOi kidney transplant recipients with or without ABMR (47). At day 10 they found different gene expression for some specific genes involved in oxidative phosphorylation, apoptosis, NK cells and complement between the 2 groups. PBMC transcriptome analysis was sufficient to differentiate patients with an accommodated graft from those with early ABMR. Thus, accommodation in the ABOi setting mainly involves changes in the graft endothelium, especially with the expression of complement regulatory proteins, but also changes in the recipient immune response. Other mechanisms of graft acceptance have been or are being explored (reduction of blood group antigen levels in the graft (48, 49), B-cell tolerance…) (30). Data from other organs ABOi transplantation are relatively scarce although accommodation situations have been reported in heart and liver transplantations as well (50–52).

### Xenotransplantation

Xenotransplantation refers to transplantation between two different species. Despite several attempts of clinical xenotransplantation in humans performed since the nineteenth century, the activity remains mostly at a research level, essentially with pig-to-non-human primate (NHP) models (53). Xenotransplantation can be described as concordant (between two phylogenetically closely related species such as mouse to rat or non-human primate to human) or discordant (not closely related species as pig to human). Pigs are the preferred animals for xenotransplantation in humans because of a close anatomy and physiology (54).The immunological barrier between pigs and humans/NHP is the major hurdle of xenotransplantation, but other issues, such as the transmission of infectious agents and regulatory and ethical aspects are also of major concern.

Transplantation of a wild-type pig organ to NHP without treatment leads to hyperacute rejection (HAR), in a time range of a few minutes to hours (55). This is observed only in discordant combinations due to preformed xenogeneic natural antibodies (XNA) in NHP serum directed against the major xenogeneic Ag galactose-α-1,3-galactose (Gal). Gal is expressed by all mammalians except Old World NHP, large monkeys and humans. Acute humoral xenograft rejection (AHXR) is also triggered by the antibody response toward the xenograft and shares features with allogenic ABMR. AHXR involves recipient posttransplant elicited XAb. IgG and IgM XAb that cause damages to the transplant endothelium by CDC and ADCC, inflammation and endothelial activation. *In vivo*, accommodation was essentially observed in various xenogeneic concordant models, but not really described in true xenogeneic discordant combinations.

#### Concordant models

Bach et al. studied accommodation in a xenogeneic concordant hamster-to-rat heart transplant model (56–58). Adding a complement inhibitor (cobra venom factor (CVF)) to conventional IS resulted in a prolonged allograft survival in 75% of recipients (56). Deposition of Ig in the graft was found in both rejected hearts and long-term surviving grafts. These long-term surviving grafts despite XAb were considered “accommodated”. Analyses of graft biopsies suggested that accommodation was associated with the up-regulation of protective genes known to prevent apoptosis, such as HO-1, A20, Bcl-2 and Bcl-xL in ECs and smooth muscle cells (SMCs), with a Th2 cellular response and a difference in IgG subtypes with a predominance of IgG2c (poorly fixing the complement) deposition. Serum transfer from heart recipients carrying an accommodated heart into naïve heart recipients leaded to HAR and transplantation of an accommodated heart into a recipient of a previous heart exhibited long-term survival (58). In the same model, splenectomy performed before or up to two days after xenotransplantation resulted in long-term heart survival in cyclosporin A (CsA)-treated rats (59). This was associated with an abrogated or delayed and decreased anti-donor IgM response. Histological analyses of surviving xenografts in CsA-treated rats with splenectomy exhibited normal cardiac histology, with no IgM deposition and expression of protective molecules (HO-1, A20) on ECs and SMCs. Thus, low levels of XAb IgM seem to promote the expression of protective genes in the graft, participating in building an accommodated phenotype. Modulating the immunosuppression with a short duration of leflunomide treatment addition to CsA maintenance therapy in a similar model allowed prolonged graft survival associated with the an inhibition of the T-independent XAb production as well as a controlled T-dependent response both in a donor species-specific manner (57). Finally, the situation of a presensitized recipient exhibiting non-natural anti-donor IgM and IgG in a concordant model was explored (60). The use of CVF/CsA prevented HAR, but not the development of AHXR. Blood exchange combined to CVF/CsA/cyclophosphamide and splenectomy promoted long-term survival of xenografts, despite presence of XAb (with an attenuated rebound) and complement. Surviving hearts also exhibited the expression of protective genes in ECs and SMCs and an intragraft Th2 response. Thus, in concordant models, accommodation defined as graft survival despite XAb appears to involve changes in the immune response (notably different Ig subclasses) and local changes in the endothelium, triggered by low XAb titers, that make it resistant to XAb toxicity.

#### Discordant models.

Although accommodation has not been demonstrated *in vivo* in discordant combinations such as pig-to NHP models, some advances were made in controlling the humoral xenogeneic response involving the control of XNA production/fixation and complement activation with strategies combining IS treatments and the use of genetically modified donors. The use of organs from pigs transgenic for human CD55 allowed long-term graft survival in NHP up to three months (61–65). Although the use of such transgenic organs prevented xenograft HAR in NHP, recipients developed AHXR despite associated IS treatment. A second innovative strategy was to abrogate the expression of the major xenogeneic epitope, Gal, by engineering GT-KO pigs (66–68). No HAR occurred in NHP transplanted with GT-KO pig organs. Finally, several targets of gene engineering were combined, associating knock-out for GT and transgenic expression of human complement regulatory proteins [e.g. GTKO, hCD46 (69)] and thromboregulatory molecules such as thrombomodulin [e.g. GTKO, hCD46, hTMB (70, 71)]. In a model of heterotopic heart transplantation in baboons, a prolonged survival of GTKO, hCD46 and GTKO, hCD46, hTMB xenografts was observed up to 8 months and up to 31 months respectively (69, 70). In both studies, Ab remained low under IS treatment and increased at rejection. Moreover, long-term survival of GTKO, hCD46, hTMB xenograft was achieved for the first time in a life-supporting cardiac xenotransplantation model in baboons up to 195 days (71). No anti-nonGal Ab neither complement deposition were observed in rejected grafts, and the anti-donor humoral response of the recipient was abolished in long survivor recipients (71). The use of continuous hypothermic perfusion of heart prior to transplantation brings some advantages in term of cardiac function, oxygen supply, prevention of early graft failure in comparison to their static ischemic preservation and may rely on reduced endothelial damages (72). In these two recent *in vivo* studies, long-term survival of genetically modified xenografts in presence of low titers of post-transplantation anti-nonGal Ab could suggest an induction of accommodation. However, further investigations are needed in these models to validate this hypothesis.

Indeed, *in vitro* studies suggest that accommodation may occur in presence of low titers of XNA. Incubation of porcine ECs with low titers of XNA was associated with a decreased cytotoxicity of XNA and a decreased expression of VCAM and MHC class I molecules or an increased expression of HO-1 by porcine ECs (59, 73, 74). In an *in vivo* model of pig-to-pig kidney transplantation from wild type pigs (expressing Gal) to SLA-matched GT-KO pigs exhibiting anti-Gal XNA under FK506 treatment (75), Gal positive kidneys were rejected at earlier time points in aged GT-KO recipients compared to younger ones (increasing titers of cytotoxic IgM and IgG XNA with their age). One graft was accommodated in a young recipient exhibiting persistent IgM cytotoxic XNA and a rebound of IgG XNA greater than baseline after an initial posttransplant decrease. Graft accommodation was also achieved in an older recipient who underwent two pretransplant plasmaphereses, reducing but not eliminating the pretransplant IgM/G anti-Gal Ab titers. Histological analysis of accommodated grafts biopsies showed IgM/G, C3 and C5b-9 deposition one hour posttransplant, but no more C5b-9 deposition in later post-transplantation period, although C3 deposition remained, associated with an up-regulation of CD59 expression. Overall, these *in vitro* and *in vivo* data suggest that accommodation of xenogeneic grafts in discordant models could be obtained and underline the importance of XNA titers in this setting.

## Accommodation in the context of allo-immunization against HLA

### The impact of DSA *in vivo*

Presence of preformed DSA before transplantation is broadly considered to have a negative impact on graft outcome. Graft survival is lower among patients with preexisting HLA-DSA (but negative lymphocytotoxic cross-match (CM) test on the day of transplantation) compared with both sensitized patients without HLA-DSA and non-sensitized patients (12). Preformed anti-HLA class II DSA seem to confer a worse prognosis than anti-class I DSA (76). *De novo* DSA appearance post-transplantation has also a negative impact on graft function (10, 77). *De novo* DSA directed against either class of HLA antigens are deleterious but DSAs directed against HLA class II antigens seem to be overrepresented in late-onset ABMR with reduced graft survival (78). Graft outcome seems to be more severely impacted by *de novo* DSA than by preformed DSA in patients with ABMR (79).

Histopathological features of ABMR are described in the Banff classification (80). ABMR can be hyperacute (in the first hours after transplantation, usually with a huge load of preformed DSA), acute or chronic. Features of antibody-mediated rejection include the presence of DSA (against HLA or other antigens), signs of complement activation on transplant biopsy (e.g. C4d deposition) and microvascular inflammation (immune cell infiltration (mainly macrophages and Natural Killer cells)) in peritubular capillaries and glomeruli (15). The key feature of ABMR diagnosis is microvascular inflammation. In chronic forms of ABMR, we observe signs of chronic endothelial damage such as transplant glomerulopathy and arteriosclerosis. The development of transcriptomic analysis has shed light on transcripts associated with allograft rejection, with a specific signature of NK cells and ECs, underlining their role in ABMR (81, 82).

As already mentioned, the transplant endothelium constitutes the first interface between the recipient’s blood and the donor tissue; recipient DSA damage the graft endothelial cells by CDC, ADCC and direct effect involving activation and proliferation of ECs (15–17). Characteristics of DSA (titer, isotype, subclass…) influence the mechanisms and the phenotype of ABMR (acute vs subclinical ABMR). Acute ABMR occurring in patients with early DSA appearance (first posttransplant year) was mainly driven by IgG3 (complement-binding subclass) immunodominant DSA whereas subclinical ABMR was driven by IgG4 immunodominant DSA (83). C1q-binding immunodominant DSA were independently associated with allograft failure. On the other hand, IgG4 (non-complement binding) DSA were associated with allograft nephropathy and FIAT lesions. Long term persistence of non-C1q binding DSA impacts allograft survival, underlying the importance of complement-independent mechanisms in the development of chronic ABMR (84). Overall, there is a continuum between DSA occurrence, ABMR and allograft loss (85).

In an analogy to ABOi transplantation, desensitization strategies are used to achieve negative lymphocytotoxicity CM in highly sensitized recipients and pursue transplantation. Transplantation of highly sensitized patients with HLA-incompatible transplant after desensitization protocol (plasmapheresis and intravenous immunoglobulins) confers a strong patient survival benefit compared with highly sensitized patients who continue to receive dialysis or undergo HLA-compatible transplant (longer waiting time) (86). A new technique to eliminate DSA is the use of IdeS (IgG-degrading enzyme derived from Streptococcus pyogenes), an endopeptidase that cleaves human IgG into F(ab′)2 and Fc fragments, inhibiting both CDC and ADCC (87). However, despite desensitization protocols, outcomes of HLA-incompatible transplantation are still inferior to HLA-compatible transplantation, which clearly is a different situation from ABOi transplantation.

In cohort studies, some patients with *de novo* DSA do not have clinical consequences. Indeed, Wiebe et al. described a group of patients (called “subclinical *de novo* DSA”) with no decrease in kidney function or proteinuria within 2 months of DSA detection. These patients had a better prognosis in terms of kidney function at 3 years and kidney graft survival compared to patients with clinical DSA (10). However, the *de novo* DSA phenotype (clinical vs subclinical) was not a predictor of allograft failure in multivariate analyses. Lefaucheur et al. showed that in patients with *de novo* DSA detection during the first year post-transplant, 40.8% had a concomitant acute ABMR, 28.8% subclinical ABMR and, 30.4% were ABMR free at the time of DSA detection (83). Loupy et al. showed that half of the patients with preformed DSA did not have clinical or subclinical ABMR at one year posttransplant (88). In a cohort of kidney transplant recipients with preexisting DSA who underwent a desensitization protocol, 20 to 30% of recipients were found to have antibody and complement deposition without histologic signs of humoral rejection (89). Aubert et al. found no correlation between low level-DSA (negative CM) before transplantation and rejection or graft outcome, suggesting the possible occurrence of accommodation (90).

Few studies tried to detect accommodation and explore its mechanisms in clinical situations of transplantation with DSA. Salama et al. described seven highly sensitized patients who received a kidney transplant after a desensitization protocol based on immunoadsorption (negative CM) (91). Four transplants were still functioning (follow-up between 4 months and 7 years) despite the return of antibodies in 3 of these 4 patients (accommodated transplants). IgG and C3 deposition were found on the endothelium in transplant biopsies of two patients with DSA return without rejection. Bcl-xL staining was positive in glomerular capillary loops and peritubular capillaries of 3/4 patients who had DSA return (two without rejection, one with chronic rejection). However, neither Bcl-2 nor HO-1 expression changed in the accommodated transplants. Jin et al. studied Bcl-2 staining in cardiac transplant biopsies from 7 recipients diagnosed with ABMR and 6 recipients without ABMR (92). Positive Bcl-2 staining on graft capillary ECs was found in 5/7 cardiac biopsies from patients developing ABMR. These 5 patients had anti-class I Ab, whereas the two patients with ABMR but negative Bcl-2 staining had anti-class II Ab, underlying a probable causal effect of anti-class I Ab in the induction of Bcl-2 expression. Only 1/6 biopsy showed a positive Bcl-2 staining on graft endothelium in patients without ABMR. These two studies suggest that exposure of the endothelium to anti-HLA Ab induces intra-cellular changes and the upregulation of cell survival genes. However, the latter do not seem sufficient to stop the rejection process. Overall, data supporting accommodation *in vivo* are scarce.

### DSA binding on recipient ECs: mechanisms of toxicity

Independently of its capacities to activate the complement and to recruit immune cells, anti-HLA antibody binding on ECs participates in allograft injury (93).

**Figure 1:**
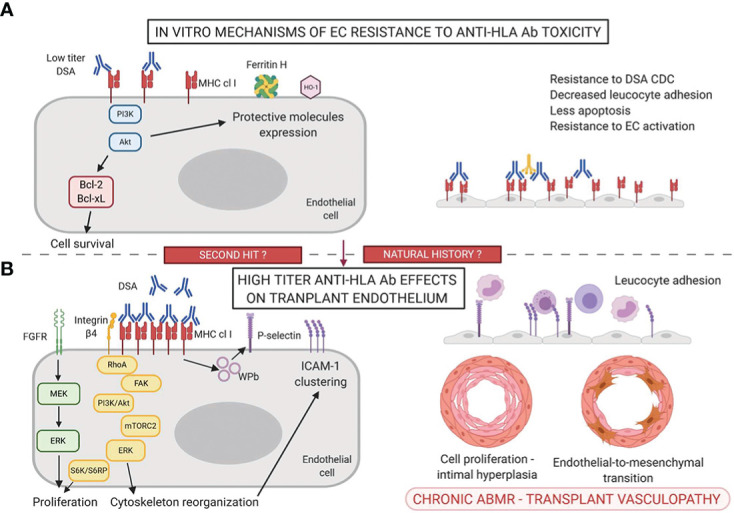
Mechanisms of accommodation **(A)** and antibody-mediated toxicity **(B)** in the anti-HLA Ab setting.

Anti-HLA class I (HLA-I) Ab induce molecular changes in ECs and SMCs of the graft that promote cell proliferation. Two important parameters of the anti-HLA-I Ab effect on ECs are the titer or concentration of the Ab and the level of HLA expression, that will influence the degree of molecular aggregation at the EC cell surface (16). High concentration of monoclonal anti-HLA-I Ab (W6/32) induces the highest degree of EC proliferation *in vitro*. Cell proliferation is induced by several mechanisms including the upregulation of the expression of fibroblast growth factor receptor (allowing binding of its ligand FGF, promoting proliferation *via* the ERK/MAP kinase pathway), the upregulation of pro-survival genes and anti-apoptotic proteins in ECs and the implication of the mTOR pathway (**Figure 1B**) (92, 94, 95). Stimulation of human aortic endothelial cells (HAECs) with monoclonal anti-HLA Ab or its F(ab’)2 fragment cross-links HLA-I molecules and stimulates the formation of a complex between HLA-I and the integrin β4 subunit (cis interactions) (96). This complex formation appears to be mandatory for induction of HLA-I mediated cell proliferation and migration, with a signaling pathway involving FAK, Src, Akt and ERK (97, 98). Jindra et al. confirmed the involvement of MHC class I signaling in the pathogenesis of ABMR in a vascularized heterotopic cardiac allograft murine model (99). Infusion of DSA was correlated with the activation of Akt and S6K involved in MHC class I cell proliferation and survival.

In the human kidney, the endothelium of glomerular and peritubular capillaries constitutively expresses MHC class II DR molecules and to a lower extent DQ and DP molecules whereas the endothelium of larger renal blood vessels does not express class II molecules (100). MHC class II expression is controlled by a specific transcription factor called CIITA (Class II Major Histocompatibility Complex Transactivator). In the pathologic setting of a transplanted kidney with acute cellular and vascular rejection, DQ is strongly induced on peritubular ECs. Renal microvascular ECs express a high level of HLA-DR at steady state whereas the addition of gamma interferon (IFNγ) is necessary to induce HLA class II (HLA-II) expression *in vitro (* 100, 101). Moreover, HLA-DR and HLA-DQ expression relies on differential transcriptional and epigenetic regulation, adding complexity to MHC class II expression (102). Given their ability to express HLA-II molecules, microvascular ECs within transplants are potential inducers and targets of *de novo* DSA. Similarly to HLA-I, ligation of anti-HLA-II Ab on HLA-II molecules induces intracellular signaling. Ab ligation of class II molecules at EC surface *in vitro* stimulates EC proliferation and migration *via* Akt, MEK, ERK and mTORC2 pathways (103, 104). This effect is reproduced when using only the F(Ab’)2 fragment of anti-HLA-DR Abs (105).

These *in vitro* findings revealing the role of anti-HLA Ab signaling in ECs, independently of complement activation or monocyte recruitment, is relevant *in vivo* and could partly explain chronic allograft vasculopathy. Endomyocardial allograft biopsies of patients with evidence of ABMR showed a significant increase in expression of p-S6K and p-S6RP (a downstream target of the PI3K/Akt/mTOR pathway) in capillary ECs (106, 107). EC activation following anti-HLA-I or -II Ab ligation can lead to endothelial-to-mesenchymal transition. Markers of endothelial-to mesenchymal transition (fascin1, vimentin and heat shock protein 47) have been specifically detected in peritubular capillaries of ABMR allograft biopsies, associated with DSA, ABMR histological criteria and were predictive of late allograft loss (108, 109). These markers were closely correlated to anti-HLA-II DSA DQ and DR, discriminating the prognosis of patients with DSA.

Finally, ECs are non-professional antigen-presenting cells (APCs) but they promote allo-recognition, leukocyte recruitment in the presence of DSA and allo-immunity modulation (110). Indeed, anti-HLA-I Ab binding at the EC surface increases adherence of monocytes to ECs *via* the expression of P-selectin (111). *In vitro* treatment of ECs with anti-HLA-I Ab triggers ICAM-1 clustering, promoting the firm adhesion of leukocytes to the endothelium (112). Anti-HLA DR and DQ DSA trigger IL-6 production by ECs, favoring a pro-inflammatory environment (105, 113).

Overall, besides CDC and ADCC, DSA at high titers have direct effects on the graft endothelium, inducing proliferation and activation of ECs, thus promoting transplant vasculopathy.

### *In vitro* models of accommodation

Few teams tried to induce an *in vitro* and *in vivo* protection of endothelial cells against DSA toxicity by incubating ECs with anti-HLA antibodies. Most of these studies focused on anti-HLA-I antibodies (**Figure 1A**, **Table 1**).

**Table 1:** In vitro induction of accommodation in anti-HLA Ab setting.

Authors, Journal, Year	Salama et al, AJT 2001	Jin et al, Human Immunology 2004	Narayaman et al, Eur J Immunol2004	Narayaman et al, Transplant Immunology 2006	Bharat et al, Transplantation 2007		Iwasaki et al, Transplantation 2012	Iwasaki et al, Transpl Int 2013
Species	Human	Human	Human	Human	Mouse	Human	Human	Human
Anti-MHC Ab	Total IgG from immunized patients(containing anti-Class I and II HLA Ab)	Anti-HLA class I monoclonal Ab(clone W6/32), full or Fab fragment	Polyclonal anti HLA-A2 Ab from 4 immunized patients	Anti-HLA class I monoclonal Ab (clone W6/32)	Anti-H2b monoclonal Ab	Anti-HLA class Imonoclonal Ab (clone W6/32)	Anti-HLA class I monoclonal Ab (clone W6/32)	Anti-HLA class I monoclonal Ab (clone W6/32)
Endothelial cell type	HUVEC	HAEC	HAEC (HLA A2)	HAEC (HLA A2)	Islets	Islets	EA.hy926 (HUVEC cell line)	EA.hy926 (HUVEC cell line)
Saturating/High dose	1/5- 1/7 dilution	10 µg/mL	6000 ng/mL	10 000 ng/mL	1-10 µg/mL	1-10 µg/mL	10 µg/mL	10 µg/mL
Subsaturating/low dose	1/100- 1/200 dilution	0.1 µg/mL	60 ng/mL	100 ng/mL	1-100 ng/mL	1-100 ng/mL	1 µg/mL	1 µg/mL
other molecules								Complement 1%Thrombin 0.5 U/mLResveratrol 50 µM
Incubation time	5 days (functionnal test and phenotype)	3 days (phenotype), 10 min (signalling)	3 days (functionnal test, apoptose assays, phenotype), 15 min(signalling)	3 days (functionnal test, phenotype), 15 min (WB)	48 h	48 h	24 h (functional test, and phenotype), 1 h (signalling)	24 h Resveratrol +/- followed by 24 h W6/32 +/- complement and thrombin (functional test, phenotype),2 h (signalling)
Accommodating dose	Anti HLA Ab subsaturating dose	W6/32 subsaturating dose	Anti HLA Ab subsaturating dose	W6/32 subsaturating dose	Anti-H2b Ab subsaturating dose	W6/32subsaturating dose	W6/32 subsaturating dose	W6/32 subsaturating dose and Resveratrol 50 µM
Functionnal test	CDC (LDH release) after exposition to saturating dose of IgG		* CDC (^51^Cr release) after 1h expostion to saturating dose of anti-HLA A2 Ab *PBL adhesion after 1h exposition to saturating dose of anti-HLA A2 Ab * High doseanti-HLA A2 Ab + complement mediated apoptosis (TUNEL assay and Caspase 3 assay (fluorometry))	* CDC (^51^Cr release) after 1h expostion to saturating dose of anti-HLA A2 Ab *High dose W6/32 + complement mediated apoptosis (TUNEL assay and Caspase 3 assay (fluorometry))	see table *in vivo*		CDC after 1h exposition to saturating dose of W6/32 (MTT)	CDC after 1h exposition to saturating dose of W6/32 (MTT)
Result of functional test	Resistance to CDC		* Resistance to CDCInhibition of PBL adhesion to ECfewer apoptotic cells * lower caspase 3 activity	* Resistance to CDC* fewer apoptotic cells * lower caspase 3 activity			Resistance to CDC	Complement and thrombin each inhibit the protecting effect of W6/32 at subsaturating dosis against saturating dose W6/32 CDCResveratrol induces EC resistance to W6/32 CDC and restores the protection against W6/32 saturating dose CDC in the presence ofW6/32 subsaturating dose, complement 1% and thrombin 0,5 U/mL
Signaling pathways		↗ Akt phosphorylation via Scr, PTK, FAK and PI3K (Fab and full Ab idem)	↗ Bad and Akt phophorylation	* ↗ Bad, Akt, PI3K, PDK1 and PKA phophorylation* ↗ production of cAMP			↗ Akt phosphorylation	* ↗ AMPKα phosphorylation, ↘ Akt and S6K phosphorylation, no effect on ERK* The protective effect of Resveratrol is AMPKα- dependant (abolished by siAMPKα)
Transcriptionnal changes					↗ Bcl-2	↗ Bcl-2	↗ Ferritin H and HO-1	* ↗ Ferritin H, HO-1 and KLF2 (Resveratrol)* ↗ Ferritin H and HO-1 (Resveratrol + W6/32 subsaturating dose + Complement + thrombin)
Phenotypical changes	* ↗ Bcl-xL* Resistance to the upregulation of ICAM-1 induced by saturating doses of IgG (FC)	* ↗ Bcl-2 and Bcl-xL* ↗ Bad phosphorylation (Fab and full Ab idem)	* ICAM and VCAM downregulation* HLA-A2: no change* ↗Bcl-xL, Bcl-2 and HO-1	Resistance to TNF-α (no induction of ICAM-1/VCAM-1)* HLA-A2: no change↗ Bcl-xL, Bcl-2 and HO-1		↗ HO-1, Bcl-xL and Bcl-2 in islets endothelial cells		↗ CD55 and CD59 (Resveratrol)

* was usedto mark items when there was more than one idea/results in a box. MHC, major histocompatibility antigen; HLA, human leukocyte antigen; Ab, antibody; HUVEC, human umbilical vein endothelial cells; HAEC, human aortic endothelial cells; WB, Western-Blot; CDC, complement-dependant cytotoxicity; PBL, peripheral blood leukocytes. Upward arrow, increase; downward arrow, decrease.

Two studies explored the accommodating effect of DSA on ECs by incubating HUVECs (human umbilical vein endothelial cells) or HAECs with total IgG from sensitized patients (91, 114). Both studies showed a reduction of high dose DSA CDC following a pre-incubation of ECs with low doses of DSA. Furthermore, Narayanan et al. showed that incubation of ECs with low concentrations of DSA protected them from apoptosis and from peripheral blood lymphocytes adhesion, consistent with relatively low expression of adhesion molecules ICAM and VCAM. After exposure of ECs to low doses of DSA, intracellular signaling was modified with an increase of Bad and Akt phosphorylation (critical step in PI3K-dependent cell survival) and the expression of the anti-apoptotic molecules Bcl-xL, Bcl-2 and of the anti-inflammatory gene HO-1 was enhanced.

Besides the use of anti-HLA allo-Ab, several experiments were also conducted on human ECs using the W6/32 anti-class I monoclonal Ab (44, 92, 115, 116). ECs pre-incubated with sub-saturating concentrations of W6/32 for one or three days owing to the study were partially resistant to the CDC of saturating doses of W6/32. Furthermore, Narayanan et al. showed fewer apoptotic cells after incubation with low doses of W6/32. Incubation of ECs with low doses of W6/32 led to an increased expression of the anti-apoptotic molecules Bcl-xL and Bcl-2, the cytoprotective molecules HO-1 (a stress responsive enzyme that catabolizes free heme (117)) and Ferritine H (an intracellular molecule with anti-oxydant properties (118)) and prevented the up-regulation of the adhesion molecules ICAM-1 and VCAM-1. Iwasaki et al. found no change in the expression of complement regulatory proteins CD55 and CD59. Regarding intracellular signaling pathways, the three teams showed a phosphorylation of Akt and PI3K with low doses of W6/32, a pathway implicated in cell survival. Jin et al. highlighted the involvement of Src and FAK in class-I mediated phosphorylation of Akt and PI3K. Bcl-2 but not Bcl-xL was shown to be a downstream target of the PI3K/Akt pathway. Narayanan et al. showed an increase in Bad and PKA phosphorylation and cAMP production after exposure to low doses of W6/32. Treatment with PI3K and PKA inhibitors completely reversed this protective effect, pleading for a combined role of PI3K and PKA pathways in EC resistance to CDC induced by sub-saturating doses of anti-HLA Ab. Ligation of anti-HLA-I antibodies also induces the over-expression of the transcription factor Nrf2 *via* activation of PI3K/Akt (119). Nrf2 binds to Ferritine H Anti-oxidant Responsive Element (ARE), thus activating the transcription of the Ferritin H gene. Overexpression of Bcl-2 and Bcl-xL and phosphorylation of Akt were similarly induced by W6/32 F(ab’)_2_ fragment and by total Ab, excluding a signaling by Fc receptors (92). Addition of thrombin and complement *in vitro* abolished the protecting effect of low dose W6/32 against Ab/complement-mediated cytotoxicity (45). Complement, but not thrombin, inhibited anti-HLA mediated cytoprotective gene expression (Ferritin H and HO-1). These complex features appear as limiting factors for the induction of accommodation *in vivo*. Overall, results obtained with W6/32 reproduce those obtained with DSA.

### Animal models of accommodation

In addition to *in vitro* cellular studies, accommodation induction was also studied *in vivo* with two different strategies: graft or donor pre-treatment with anti-MHC Abs and the use of complement inhibitors in the early posttransplant period (**Table 2**).

**Table 2:** In vivo induction of accommodation in allogeinic transplantation.

Authors, Journal, Year	Bharat et al, Transplantation 2007	Fukami et al, Transplantation 2012	Wang et al, JI 2007	Rother et al, AJT 2008	Chen et al, AJT 2011
Species	Mouse	C57BL/6 transgenic mice (HLA-A2)	Mouse	Mouse	Non Human Primate
Organ	Islets	Heart	Heart	Kidney	Kidney
Agent to obtainaccommodation	Anti-HLA Ab subsaturating dose pre-incubation	Anti-HLA Ab subsaturating dose pre-incubation	Complement inhibition during early postTx period	Complement inhibition during early postTx period	Complement inhibition during early postTx period
Protocol	Transplantation of ex vivo accommodated islets (Anti H2b Ab subsaturating dose 48 h) to a full MHC- mismatch sensitized recipient	Heterotopic heart transplantation from HLA-A2 Tg mice treated with W6/32 subsaturating dose (50 µg ip) 48 h before Tx to non transgenic sensitized mice	Allogeneic heart transplantation from C3H (H-2^k^) donor to recipient BALB/c (H- 2^d^) mouse presensitized with C3H skin graft	Allogeneic kidney transplantation from C3H (H-2k) donor to recipient BALB/c (H- 2d) mouse presensitized with C3H skin graft	Allogeneic kidney transplantation to recipients pre-sensitized with donor skin
Immunosuppression	No IS	No or rabbit anti-mouse lymphocyte serum (ALS)	CsA + anti-C5 mAb or CsA + CyP (2 days)/anti-C5 mAb (60 days). Controls no IS or monotherapy	CsA + LF10-0195 (14 days) + anti-C5 mAb (60 days). Controls no IS or monotherapy	Long-term triple therapy CsA + MPA + Cs+ CVF 14 days. Controls CsA+ MPA + Cs
Graft survival	Prolonged survival (6 vs 1 day) for accommodated grafts	Prolonged transplant survival of hearts from donors pre-treated with W6/32 50 µg (pre-treatment + ALS 25 vs Pre-treatment 15 vs no treatement 5 days)	Mean graft survival controls 3.1 days, CsA+ Anti-C5 mAb 11.9 days, CsA + CyP indefinite survival	Mean graft survival controls 8.5 days, CsA+ LF + Anti-C5 mAb indefinite survival	Controls graft failure between day 2 and 4, CVF group indefinite graft survival (> 715 days) with normal renal function
Graft histological analysis		Controls (isotype) AMR Day 5, Treatment with W6/32 cellular rejection Day 15	Controls features of AMR, CsA + anti-C5 mAb AMR, CsA + CyP + anti-C5 mAb normal histology, C3, C5, IgG and IgMdeposits.	controls AMR + CMR, triple therapy no AMR no CMR, mild C5 deposition, C3 deposition	controls AMR +/- ACR, CVF normal histology, IgG deposits, low C3c, low/mild C4d and low C5b-9 deposits
Graft proteic changes		* ↘ expression of ICAM-1, VCAM-1, PECAM (Day 5) * ↘IL-1β, TNF-α, IL-6, and IL-12	↗ Bcl-2 and Bcl-xL	↗ Bcl-xL and A-20 (D11 and D100)	↗ Bcl-2, CD59, CD46
Graft RNA changes		* ↗ Bcl-2 and HO-1 * ↗ Bcl-xL, Bcl-2, HO-1, Survivin (day 5)			
Recipient changes			Switch from IgG2a to IgG2b in recipients of accommodated hearts serum	Lower anti-donor IgG and IgM MFI at day 100 in triple therapy groupIgG2b predominance in recipients of triple therapy group	Long survivors: persistent but low levels of anti-donor IgG
Additionnal results		Major role of Bcl-2 in graft protection (rejection day 9 after Bcl-2 silencing by shRNA)	accommodated heart grafts retransplanted into presensitized recipients with accommodated primary heart grafts (CsA) exhibit indefinite survival but not when the second heart or the recipient is not accommodated		

* was usedto mark items when there was more than one idea/results in a box. MHC, major histocompatibility antigen; HLA, human leukocyte antigen; Ab, antibody; HUVEC, human umbilical vein endothelial cells; HAEC, human aortic endothelial cells; WB, Western-Blot; CDC, complement-dependant cytotoxicity; PBL, peripheral blood leukocytes. Upward arrow, increase; downward arrow, decrease.

Mohanakumar’s team studied accommodation in murine islets and cardiac allogeneic transplant models (120, 121). In a murine model of islet allografts into full MHC-mismatch sensitized recipients without immunosuppression, the *in vitro* pre-incubation of islets with sub-saturating concentration of monoclonal anti-MHC class I H2^b^ Ab during two days resulted in an up-regulation of the anti-apoptotic genes and proteins Bcl-2, Bcl-xl and HO-1 in islet endothelial cells. Transplantation of these *ex vivo* accommodated islets in sensitized recipients resulted in a prolonged survival without allograft complement deposition compared to untreated islets (120). The same team developed a single HLA-mismatched heterotopic murine heart transplant model (HLA-A2 transgenic mice heart into HLA A2-sensitized C57BL/6 recipients) (121). Pretreatment of HLA-A2 transgenic mice with a low concentration of W6/32 Ab (intraperitoneal injection) for two days before transplantation induced a prolongation of allograft survival (mean 15 ± 2 days) when compared to untreated organs (2 ± 1 days). Expression of various genes was analyzed in accommodated allografts on day 5 post-transplantation, resulting in i) a significant increase in the expression of anti-apoptotic genes Bcl-xL, Bcl-2, HO-1, Survivin, ii) low levels of alloAb and complement C4 graft deposition, iii) a significant decrease in the expression of the adhesion molecules ICAM-1, VCAM-1, PCAM, of the inflammatory cytokines IL-1β, TNF-α, IL-6, IL-12 and of the chemokines MCP-1, MIG, MIP-1, IL-8. Although donor pretreatment with W6/32 Ab prevented AHR in sensitized recipients, it did not prevent acute cellular rejection (ACR) occurring at day 15. These two studies show that organ or donor pre-treatment with low levels of HLA-I Ab before transplantation in highly sensitized recipients can confer protection against humoral rejection, i.e. accommodation.

Wang et al. developed a mouse model of acute ABMR in hearts transplanted into presensitized mice (122). Recipient treatment with CsA and anti-C5 mAb prolonged graft survival (mean 11.9 days vs 3.1 days) but did not prevent ABMR. On the contrary, treatment with CsA (long term), cyclophosphamide (2 days) and anti-C5 mAb (stopped at day 60) allowed indefinite heart allograft survival despite detection of anti-donor antibodies and normal complement function after anti-C5 mAb treatment interruption. Grafts at day 100 showed normal histology despite evidence of C3, C5, IgG and IgM deposition. With second grafts experiments, they demonstrated that both changes in the graft and in the recipient are required to induce prolonged graft survival since they observed the expression of BcL-2 and Bcl-xL in accommodated grafts and a switch from IgG2a to IgG2b in the serum of the recipients. The same team demonstrated the efficacy of a triple therapy of long term CsA, LF15-0195 until day 14 and anti-C5 mAb until day 60 to achieve indefinite allogeneic kidney transplant survival in presensitized mice (123). Bcl-xL and A-20 were overexpressed in accommodated grafts. Untreated mice showed a predominance of IgG1, IgG2a and IgG3 whereas recipients with accommodated grafts showed a predominance of IgG2b. The therapeutic strategy combining blockade of T-B-cell cooperation (CsA and LF15-0195) and complement prevented ABMR whereas a single treatment or combination of only two treatments failed in this purpose. The effect of complement inhibition was also studied in a kidney allograft model in NHP pre-sensitized with donor skin allografts (124). Long-term graft survival was achieved in three out of five macaques treated with an immunosuppressive regimen containing long term CsA, MPA and prednisolone associated to CVF during the first 14 days post-transplantation. Control presensitized animals treated with IS therapy without CVF experienced graft failure due to ABMR +/- ACR between two and four days post-transplantation. The long survivors showed normal allograft histology, with IgG but low C3c, low/mild C4d and low C5b-9 deposition, compared to rejected allografts. Long survivors had persistent but low levels of DSA and return of normal levels of serum C3 after CVF discontinuation. Moreover, anti-apoptotic and complement regulatory gene analyses revealed an enhanced expression of Bcl-2, CD59 and CD46 in renal biopsies of long survivors at most-time within the 100 days posttransplant, whereas the expression of Bcl-xL and CD55 was not significantly different from normal kidneys. These three last *in vivo* studies show that the temporary association of complement-blockade in the early posttransplant period with conventional immunosuppressive therapy in sensitized animals prevents ABMR and induces accommodation. Whether complement blockade itself or other mechanisms favored by the absence of complement activation induces accommodation needs to be clarified. Modification of the endothelial phenotype (expression of anti-apoptotic molecules, of complement regulatory proteins) and some modifications in IgG subtypes seem to be associated in this setting. Complement inhibition was achieved with anti-C5 mAb for 60 days in the first two studies, with CVF for 14 days in the third study. Our team studied the effect of a C1-inhibitor in allo-immunized baboons receiving a kidney transplant (125). ABMR occurrence was postponed after the discontinuation of the C1-inhibitor (5 days of treatment) but no long term survival was achieved. The association with standard IS was not studied and obviously seems to be mandatory for long term survival as described in the aforementioned studies.

## Perspectives

### Common features and discrepancies between the three settings

In all three settings (ABOi, xenotransplantation and allo-immunization), the endothelium appears to be at the center of the phenomenon. These non-professional APCs increase their immunogenicity in response to different triggers. The induction of accommodation involves the activation of intracellular signaling pathways that leads to the expression of protective molecules at the cell surface. *In vitro* and *in vivo* studies show that some mechanisms of endothelial protection against anti-A/B, XAb and anti-HLA antibodies are shared.

ABOi and xenotransplantation (discordant models) share some common features. In those two situations, the antibody response is first T-cell independent, elicited by carbohydrate antigens (ABO and Gal) carried by proteins and lipids. ABO antigens are carried by many membrane proteins at the endothelial surface of the kidney, including PECAM1, von Willebrand factor, plasmalemmal vesicle-associated protein, protein band 3 anion transporter and integrin α6 (126). Interestingly, crosslinking of these ABO bearing glycoproteins may induce survival signaling cascades as it has been described for integrin α6 and PECAM-1 (127, 128). On the other hand, HLA molecules are highly polymorphic proteins, the anti-HLA Ab and the XAb (excluding anti-Gal) production is T cell-dependent. Iwasaki et al. showed that anti-A/B Ab at saturating concentration induced the inhibition of ERK phosphorylation and the upregulation of CD55 and CD59 molecules at the endothelial surface whereas EC protection against DSA CDC was induced by incubation with low dose DSA that induced the activation of PI3K/Akt signaling pathway and HO-1 and ferritin H expression (44). This difference in the doses of Ab needed to induce accommodation and in molecular changes pleads for different mechanisms involved. The accommodation/cell proliferation balance in the anti-HLA setting seems to depend on the degree of molecular aggregation, which is determined by the specificity and concentration of the antibodies (16). The link between anti-HLA Ab level and the degree of molecular aggregation at the endothelium surface *in vivo* is difficult to evaluate.

A drawback of *in vitro* studies of accommodation in the presence of anti-HLA antibodies is the use of macrovascular endothelial cells (HUVECs or HAECs). *In vivo*, the target of these DSA is the microvascular endothelium (in the kidney glomeruli and peritubular capillaries) that displays different phenotypes and functions compared to the macrovascular one (129). Constitutive expression of HLA-II by renal microvascular cells is particularly relevant (100). The level of expression of HLA-II may also differ between individuals, constitutively and owing to different activation triggers (130). Moreover, most *in vitro* studies of accommodation induction in the presence of anti-HLA Ab focused on anti-HLA-I Ab, whereas anti-HLA-II Ab display a high pathogenicity and are strongly implicated in chronic ABMR.

Recent data highlighted intricated phenomenons at the endothelium level between anti-A/B Ab and anti-HLA Ab. Kobayashi’s team demonstrated *in vitro* that preincubating blood group A/B-expressing endothelial cells with anti-blood group A/B Ab prevented the expression of HLA-DR by these ECs after 48h of IFNγ treatment. Anti-A/B ligation on ECs upregulated complement regulatory proteins CD55 and 59 through the inactivation of ERK and mTOR pathways. Overall, anti-A/B Ab ligation induced resistance to HLA-DR Ab-mediated CDC against IFNγ-treated cells (131). Interestingly, the same team reported clinical data indicating that the incidence of DR-associated anti-class II DSA and biopsy-proven chronic ABMR were lower in ABO-incompatible renal transplant recipients than in ABO-identical or compatible recipients (132). This trend towards less anti-class II immunization after ABO-incompatible transplantation has been described by other groups in pediatric thoracic transplantation and in adult renal transplantation (133, 134).

Other mechanisms could participate in accommodation induction. Data from mice models in xeno- and allo-transplantation also suggest that accommodation is associated with a change in IgG subtypes, with a predominance of IgG2, but this has not been shown in other species (56, 122). Data from animal models in allotransplantation suggest that the control of the immune response is important to induce accommodation, in association with initial complement blockade. Endothelial chimerism with detection of recipient’s ECs in the graft has been described but does not seem to be associated with a better prognosis but rather with rejection (48, 135, 136). The level of expression of the antigen probably also influences the capacity of the endothelium to modify its phenotype to an accommodated one. Class II MHC expression on pretransplant biopsy has been shown to be correlated with a worst kidney graft function at one year (130).We can hypothesize that graft injury (from ischemia/reperfusion and infections notably) modulate class I and/or class II expression on the graft endothelium. In ABOi transplantation, blood type Ag expression in the graft appears to decrease over time but is still detectable in all grafts at ten years (48).

### Pharmacological tools for the induction of endothelial protection

As described above, protection of endothelial cells against DSA CDC *in vitro* can be induced by low doses of DSA. However, as accommodation is rarely described *in vivo* in allotransplantation, some pharmacological tools to enhance endothelial protection against DSA toxicity may be useful.

#### Complement inhibition

Activation of the complement cascade *via* the classical pathway by DSA binding on allo-antigens at the EC surface leads to acute damages and can be assessed by C4d or C5b9 staining (80, 137). Moreover, activation of the complement cascade leads to the release of split products called anaphylatoxins (C3a, C4a, C5a) that contribute to inflammation and graft damages.

In ABOi transplantation, C4d staining is positive without features of inflammation of the microcirculation, consistent with activation of the complement cascade by the ligation of anti-A/B Ab to the endothelium (40). However, some changes in the endothelium phenotype such as overexpression of complement regulatory proteins may have stopped the complement cascade. In concordant xenotransplantation, the addition of CVF (an analogue of human C3 that activates the complement cascade leading to the depletion of serum complement activity (138)) to IS regimen in the early posttransplant period has been shown to promote accommodation (56, 60). In the allo-sensitized context, *in vitro* addition of complement to low dose of DSA seems to prevent the accommodation phenomenon to occur (45). We have described above *in vivo* studies of heart or kidney allotransplantation to sensitized recipients in mice or NHP models associating conventional immunosuppression with transient early complement inhibition with CVF or anti-C5 mAb. This association prevented ABMR and allowed accommodation, a state that persisted after the interruption of complement blockade (122–124).

Complement inhibition in the early period posttransplant may also prevent or stop the deleterious aspecific complement activation occurring in the deceased donor, in the recipient before transplantation and during ischemia-reperfusion injury (43). Importantly, while DSA activate the classical pathway of the complement system, activation of the complement during ischemia-reperfusion injury (IRI) involves all three pathways.

**Figure 2:**
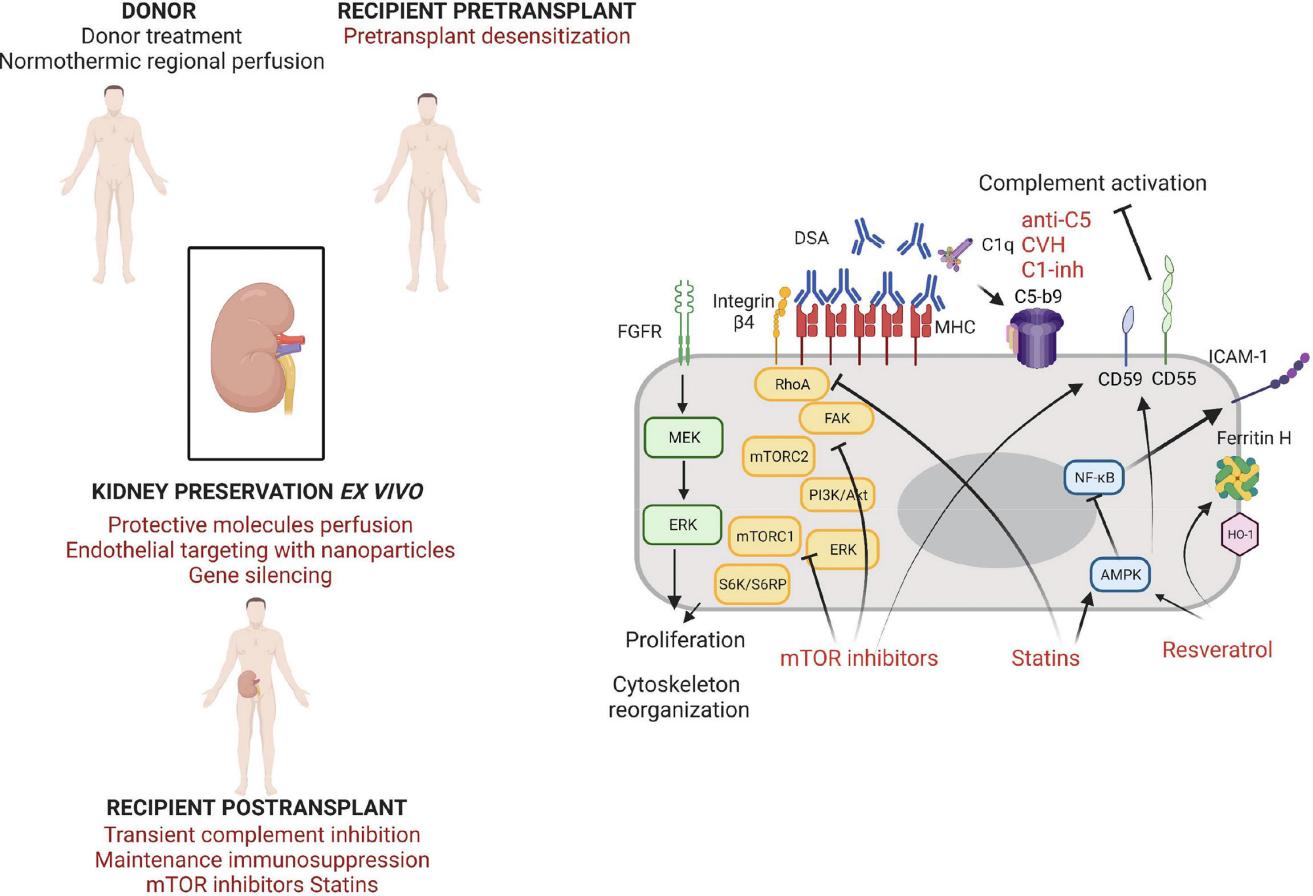
Strategies of endothelium protection in kidney transplantation.

The humanized anti-C5 mAb Eculizumab (Soliris, Alexion) and preparations of recombinant human C1 inhibitor (rhC1INH, Pharming, NL) are available in the clinics and under evaluation for the treatment of ABMR and in desensitization strategies (43). Eculizumab blocks cleavage of C5 into C5a and C5b and prevents the formation of membrane attack complex. A protective effect of a 9-week Eculizumab course in the early posttransplant period in the prevention of early ABMR in positive CM HLA incompatible kidney transplantation has recently been demonstrated (139, 140). The occurrence of accommodation following such therapeutic strategies of early complement blockade in CM positive patients needs to be investigated. Schinstock et al. reported no better long-term outcomes for Eculizumab-treated positive CM recipients compared to positive CM recipients without Eculizumab (141). Stratification of CM positive patients on DSA characteristics (better outcome for patients with negative IgG3 after treatment) may help identify patients that would benefit from such therapies (142). Indeed, Eculizumab is more efficient to abrogate ABMR in patients with complement-activating DSAs (C1q-binding DSAs) compared to patients without complement-activating DSAs (143). Eculizumab seems promising in the treatment of ABMR providing it may exert a long-term effect (144). Proximal complement inhibition with plasma C1 esterase inhibitors (C1 INH) for 14 days has been successfully tested in addition to plasmapheresis and IVIg in ABMR (145). Compared to C5 blockade, this upstream complement inhibition may be beneficial by stopping the cascade before the release of anaphylatoxins and further studies are warranted to confirm its benefits (**Figure 2**).

#### IL-6 Blockade

Targeting IL-6 is a promising approach in ABMR treatment. IL-6 plays a role in T cell maturation, germinal center formation, differentiation of naive B cells into plasma cells and in high affinity antibody production (146). Moreover, DSA-endothelial cells interaction seems associated with a local production of IL-6 by endothelial cells, that appears to take part in the injury process of AMBR and orientate locally the immune response (decrease of regulatory T cells, increase of Th17 cells) (105). IL-6/IL-6R interaction can be blocked by anti-IL-6 antibodies (clazakizumab) or anti-IL-6R (tocilizumab). Their use has been reported in small cohorts for ABMR treatment and desensitization, with mixed results (147–150). Whether the use of anti-IL-6 and anti-IL-6 receptor antibodies during desensitization or ABMR treatment will also favor local changes in endothelial cell phenotypes remains to be demonstrated.

#### Resveratrol

Resveratrol is a polyphenol naturally produced by several plants and fruits (151). Many studies have described beneficial effects in healthy and pathologic conditions, linked to its radical scavenger and antioxidant properties. Resveratrol has several effects on the endothelium, mediated by sirtuin-1, AMP-activated protein kinase and estrogen receptors (152). It enhances endothelial NO production and upregulates eNOS expression and activity by ECs and inhibits endothelin-1 synthesis by ECs and SMCs. This contributes to the reduction of the oxidative stress and to vasodilation, with *in vivo* data showing a protective effect of Resveratrol on vascular remodeling and blood pressure (153). Moreover, Resveratrol exerts a protective effect on ECs in inflammatory conditions *via* the activation of AMPK leading to the inhibition of NF-κB, resulting in decreased ICAM-1 expression and monocyte adhesion in response to TNF-α (154). Resveratrol appears to inhibit the expression of CD40 and the production of ROS by ECs treated with TNF-α, *via* the repression of the p38 MAPK/NF-κB pathway (155). Resveratrol has been suspected to explain partly the protective effect of red wine against cardio-vascular diseases (known as the French paradox) but a major drawback of *in vitro* studies is the use of Resveratrol concentrations much higher than natural concentrations of dietary compounds (151). A recent study deciphered the effects of Resveratrol at concentrations present in red wine on human coronary artery endothelial cells (156). They showed an induction of HO-1 expression, CD46 and CD55 leading to a reduced complement fragment C3b deposition, underlining a possible protective effect of Resveratrol against complement deposition that could be used for accommodation purpose. Moreover, many studies have demonstrated a protective role of Resveratrol in IRI of the kidney and of the myocardium, by reducing inflammatory responses and oxidative stress and promoting autophagy (157, 158).

Lian et al. assessed the effects of Resveratrol for the prevention of delayed xenograft rejection in a concordant hamster to rat cardiac transplantation model (159). They treated the recipients with Resveratrol after two weeks of immunosuppressive treatment associating Leflunomide and Tacrolimus. The Resveratrol treated-animals exhibited a significantly better graft survival, with histologically reduced neutrophil infiltration, and reduced T-cell responses. Iwasaki et al. assessed the effect of Resveratrol in an *in vitro* model of accommodation on human endothelial cells in the presence of anti-A/B antibodies and anti-HLA antibodies (45). They showed that 24h incubation of ECs with resveratrol induced the expression of CD55 and CD59 and the upregulation of the transcription of HO-1, Ferritin H and KLF2. Other AMPK activators (AICAR, Metformin) exerted the same effects but at a lower level. Incubation of ECs with 50 µM of Resveratrol during 24h strongly reduced the CDC of anti-A and anti-HLA Ab in an AMPK-dependent manner. Resveratrol was able to restore the protection against CDC (and thus the accommodation status) despite the presence of complement and thrombin. Thus, Resveratrol could be used to induce cytoprotective genes to promote accommodation both in the ABO incompatible setting and in the presence of anti-HLA antibodies (**Figure 2**).

#### Statins

Statins inhibit the enzyme 3-hydroy-3methylglutaryl coenzyme A (HMG-CoA) reductase and thus lower the endogenous production of cholesterol. In addition to their lipid lowering effect, statins exert specific effects on the endothelium. After heart transplantation, statins use reduces all-cause mortality and more specifically the incidence of cardiac allograft vasculopathy, a pathologic process characterized by concentric intimal layer thickening that is highly implicated in chronic allograft loss and involves both immune-mediated and non-immune mechanisms (160, 161). *De novo* DSA and rejection episodes are strongly associated with cardiac allograft vasculopathy. Protective effect of statins against cardiac allograft vasculopathy involves several mechanisms including cholesterol control, anti-inflammatory and immunomodulatory mechanisms. The ISHLT guidelines recommend the use of statins 1-2 weeks after heart transplant in all patients, regardless of cholesterol levels (162).

Statins control the redox state in the vascular endothelium by enhancing endothelial NO synthase expression and thus NO bioavailabiliy (163). Statins are able to reduce the expression of diverse adhesion molecules, cytokines and chemokines in inflammatory environments (164). This protective effect is partly mediated *via* the inhibition of the NF-κB pathway. Particularly, they inhibit the interaction between lymphocytes and ECs by binding to an allosteric site within LFA-1 blocking the interaction with ICAM-1, thus decreasing leukocyte adhesion and transmigration (165). A study in a rat model showed a potential benefit of simvastatin use on kidney IRI (166). Further with the immune-modulatory effect of statins, they are able to affect MHC class II induction by IFN-γ, a property that may be of particular interest for transplantation (167). Coupel et al. demonstrated that Simvastatin was able to prevent EC proliferation *in vitro* in response to high dose anti-HLA-I Ab ligation through inhibition of RhoA geranylgeranylation (97). Hamdulay et al. showed that incubation of ECs with atorvastatin *in vitro* led to an increased expression of CD55 *via* AMPK and CREB-dependent vasculoprotective pathways (168). Overall, the pleiotropic beneficial effect of statins to protect the ECs from immune and non-immune aggressions combined with a good tolerance profile makes it a good candidate to be tested in vascularized organ transplantation (**Figure 2**).

#### mTOR inhibitors

Mammalian target of rapamycin protein (mTOR) is a serine/threonine kinase which activity is influenced by multiple intracellular signals and forms two different complexes called mTORC1 and mTORC2. The PI3K/Akt pathway is one of the main signaling pathways that activate mTOR. Sirolimus and Everolimus, both mTOR inhibitory molecules, exert immunosuppressive effects by blocking IL-2 driven T cell proliferation (169). Moreover, mTOR inhibitors have anti-viral and anti-tumoral properties that are particularly interesting in the setting of transplantation (170, 171). However, some pro-inflammatory properties have restricted their utilization in transplant recipients. Potential beneficial effects of mTOR inhibitors on transplant endothelium have been suspected. In heart transplant recipients, Everolimus was superior to azathioprine (both combined to cyclosporine, corticosteroids and statins) at preventing cardiac allograft vasculopathy (172).

The mTOR pathway is implicated in endothelial cell biology and plays a role in EC proliferation induced by DSA ligation to HLA-I. Indeed, Jindra et al. showed *in vitro* that the knockdown of either mTORC1 and mTORC2 blocks EC proliferation in this context, suggesting that exposure of the graft endothelium to anti-HLA Abs may promote proliferation through the mTOR pathway (95). The same team showed that Everolimus is more effective than Sirolimus at antagonizing both mTORC1 and mTORC2 *in vitro* in ECs in response to Ab crosslinking of HLA-I molecules (173). Everolimus treatment prevented class-I stimulated cell migration and proliferation. Interestingly, Everolimus inhibited Akt phosphorylation (a situation thus different from the one observed in the *in vitro* models of accommodation induced by low doses of anti-HLA Ab) and also ERK phosphorylation. These data indicate a potential therapeutic effect of Everolimus in preventing chronic antibody-mediated rejection. Iwasaki et al. showed that *in vitro* treatment of HUVECs with Everolimus led to the upregulation of CD59 and KLF2 expression, but no change was observed for CD55, HO-1 and Ferritin H (45). The same team demonstrated that *in vitro* incubation of HUVECs with Everolimus in the presence of IFNγ partially inhibited the expression of HLA DR molecules at the cell surface (131). Hamdulay et al. identified a synergy between Rapamycin and Atorvastatin in the protection of the endothelium against complement mediated injury *via* an increased expression of CD55 at the EC surface (168). They produced these results *in vitro* on human endothelial cells but also *in vivo* with an enhancement of CD55 expression on the murine aortic endothelium after 48h of the combined treatment. Thus, mTOR inhibitors could be beneficial to prevent chronic AMR by several mechanisms such as lowering class II expression at the EC surface, inhibiting cell proliferation induced by Ab-mediated HLA crosslinking and increasing protective molecules expression such as complement regulatory proteins (**Figure 2**).

Protective role of HO-1 induction in kidney transplantation has been extensively studied in the context of IRI. Preconditioning with Hemin or Cobalt protoporphyrin (both HO-1 inducers) protects kidneys against IRI in animal models by reducing oxidative stress (174). The role of myeloid cells producing HO-1 is particularly interesting in this setting since they seem to promote tissue repair and promote an anti-inflammatory environnement (175, 176). Concerning the endothelium, Kinderlerer et al. have shown that HO-1 inducers hemin and cobalt protoporphyrin IX increase DAF protein expression by human ECs (177). Moreover, the overexpression of HO-1 (adenoviral-mediated) increases DAF expression and enhances protection against C3 deposition and complement mediated lysis.

#### Timing of accommodation induction

Timing of accommodation induction appears to be crucial. Presence of high titers of DSA and complement in the early postoperative period leads to ABMR. *In vivo* animal studies of accommodation induction in the context of anti-CMH immunization showed that inhibition of complement during the early post-operative period is mandatory to allow the accommodation phenomenon to occur. This transient complement inhibition may allow the modifications of both the EC phenotype and the immune response. In a clinical setting, the use of complement inhibitors early posttransplant is a possible solution as some agents are already used in hypersensitized recipients and others are currently in development. On the other hand, the use of IgG endopeptidase, an IgG-degrading enzyme derived from Streptococcus pyogenes (IdeS), cleaving human IgG intro F(ab’)2 and Fc fragments and thus inhibiting CDC and ADCC of DSA, could be beneficial for accommodation induction (87).

A complementary approach to early posttransplant recipient treatment may be treatment at the organ level with a strategy of preconditioning. Injection of protective molecules in the graft *ex vivo* presents the advantages of targeting the graft only and especially the graft endothelium and avoiding many of the limitations associated with systemic drug delivery. Recent developments in normothermic organ preservation are of particular interest in this view (**Figure 2**). *Ex vivo* normothermic perfusion allows better preservation of the organs, finer analysis of graft quality but also graft treatment *ex vivo*. Hosgood et al. developed the concept of a short period of normothermic perfusion of the kidney at the end of static cold storage with encouraging data in animal models and in clinical use, whereas Kaths et al. developed prolonged normothermic *ex vivo* kidney perfusion in porcine models (178–180). A promising strategy of nanoparticle targeting to the endothelium (with an anti-CD31 antibody) during normothermic machine perfusion of human kidneys has been described that may permit the delivery of drugs specifically to the transplant endothelium (181). Another team recently described a strategy of MHC transcripts silencing during *ex vivo* kidney perfusion in a rat model (182). They successfully delivered lentiviral vectors encoding shRNAs targeting β-2 microglobulin and the CIIT to the kidney through a sub-normothermic *ex vivo* perfusion system. Organ preconditioning gives the opportunity to prevent IRI and to influence the immune response by reducing immunogenicity of ECs and/or inducing an accommodated phenotype. Resveratrol may be delivered to the kidney *ex vivo* in a soluble form as described by Soussi et al. in a model of porcine auto-transplantation in *ex vivo* hypothermic perfusion (183). Ritschl et al. described a strategy of graft pre-conditioning by peri-operative perfusion of kidney allografts with rabbit anti-human T-lymphocyte globulin during static cold storage (184). Despite a better graft function at day 7 post-transplantation and fewer delayed graft function; however, no long-term benefit of the treatment was observed on graft function. Treatment of the donor is also a conceivable strategy, particularly in the setting of normothermic regional perfusion developed for organs from controlled donation after circulatory death (185, 186).

## Conclusions

The concept of accommodation is well recognized in ABOi transplantation but remains experimental in allotransplantation. Expression of protective molecules by the graft endothelium may be induced by low-dose DSA and favored *in vivo* by complement blockade. What happens at the endothelial scale between DSA appearance and ABMR occurrence remains hypothetical. A major hurdle remains the duration of graft protection against DSA. Continuous exposure to anti-HLA antibodies may be detrimental despite initial protection. Furthermore, other immune and non-immune events such as infections and cellular rejection may break the fine equilibrium between DSA and transplant endothelium by activating ECs or recruiting immune cells into the graft. Exploration of potential endothelium-protective drugs is of major interest, aiming at reducing chronic ABMR morbidity.

## Author contributions

DK and SLB-B drafted the manuscript. DK, SLB-B, SB and GB reviewed the manuscript.

## Conflict of interest

The authors declare that the research was conducted in the absence of any commercial or financial relationships that could be construed as a potential conflict of interest.

## Publisher’s note

All claims expressed in this article are solely those of the authors and do not necessarily represent those of their affiliated organizations, or those of the publisher, the editors and the reviewers. Any product that may be evaluated in this article, or claim that may be made by its manufacturer, is not guaranteed or endorsed by the publisher.

## Glossary

**Table:**

Ab	antibodies
ABMR	Antibody-mediated Rejection
ABOi	ABO-incompatible
ACR	acute cellular rejection
ADCC	Antibody-dependent-cell-mediated cytotoxicity
AHR	acute humoral rejection
AlloAb	allo-antibodies
AMPK	AMP-activated protein kinase
APCs	antigen presenting cells
CDC	Complement-dependant cytotoxicity
CM	cross-match
CsA	cyclosporin A
CVF	cobra venom factor
DSA	donor-specific antibodies
ECs	endothelial cellsGT-KO knock-down of galactosyl-transferase (enzyme synthetizing the Gal epitope)
Gal:	galactose-α-1, 3-galactose
HAECs	human aortic endothelial cells
HAR	Hyperacute rejection
HO-1	heme oxygenase-1
HLA	human leukocyte antigens
HUVECs	human umbilical vein endothelial cells
IdeS	IgG-degrading enzyme derived from Streptococcus pyogenes
IFNγ	gamma interferon
iNOS	inducible nitric oxide synthase
IRI	ischemia reperfusion injury
IS	immunosuppression
MFI	mean fluorescence intensity
MHC	major histocompatibility complex
MPA	Mycophenolic acid
NHP	Non-human primates
PAECs	porcine aortic endothelial cells
PBMCs	peripheral blood mononuclear cells
PI3K	phosphatidylinositol 3-kinase
PRA	Panel-reactive antibody
SLA	Swine Leucocyte Antigens (Swine MHC)
SMC	Smooth muscle cells
XAb	xenogeneic antibodies
XNA	xenogeneic natural antibodies
